# Targeting RAF Isoforms and Tumor Microenvironments in RAS or BRAF Mutant Colorectal Cancers with SJ-C1044 for Anti-Tumor Activity

**DOI:** 10.3390/cimb45070371

**Published:** 2023-07-13

**Authors:** Sungpyo Hong, Myeongjin Jeon, Jeonghee Kwon, Hanbyeol Park, Goeun Lee, Kilwon Kim, Soonkil Ahn

**Affiliations:** 1 Institute for New Drug Development, Division of Life Sciences, Incheon National University, Incheon 22012, Republic of Korea; hong.sungpyo@gmail.com (S.H.);; 2 Research Center, Samjin Pharm. Co., Ltd., Seoul 07794, Republic of Korea; mjjeon@samjinpharm.co.kr (M.J.); gelee@samjinpharm.co.kr (G.L.)

**Keywords:** colorectal cancer, KRAS, pan-RAF, CSF1R, tumor-associated macrophages

## Abstract

Colorectal cancer (CRC) is a significant global health issue characterized by a high prevalence of KRAS gene mutations. The RAS/MAPK pathway, involving KRAS, plays a crucial role in CRC progression. Although some RAS inhibitors have been approved, their efficacy in CRC is limited. To overcome these limitations, pan-RAF inhibitors targeting A-Raf, B-Raf, and C-Raf have emerged as promising therapeutic strategies. However, resistance to RAF inhibition and the presence of an immunosuppressive tumor microenvironment (TME) pose additional obstacles to effective therapy. Here, we evaluated the potential of a novel pan-RAF inhibitor, SJ-C1044, for targeting mutant KRAS-mediated signaling and inhibiting CRC cell proliferation. Notably, SJ-C1044 also exhibited inhibitory effects on immunokinases, specifically, CSF1R, VEGFR2, and TIE2, which play crucial roles in immune suppression. SJ-C1044 demonstrated potent antitumor activity in xenograft models of CRC harboring KRAS or BRAF mutations. Importantly, treatment with SJ-C1044 resulted in increased infiltration of T cells and reduced presence of tumor-associated macrophages and regulatory T cells within the TME. Thus, SJ-C1044 shows immunomodulatory potential and the ability to enhance antitumor responses. The study underscores the therapeutic potential of SJ-C1044 as a novel pan-RAF inhibitor capable of targeting oncogenic signaling pathways and overcoming immune suppression in CRC.

## 1. Introduction

Colorectal cancer (CRC) is a significant health concern as the third most frequently diagnosed and second most deadly cancer worldwide [1]. In CRC, the mitogen-activated protein kinase (RAS/MAPK) pathway plays an integral role. Specifically, KRAS is one of the most widely mutated oncogenes in CRC, with almost 40% of CRC patients containing activating mutations in *KRAS*. Specifically, the majority of these mutations occur at codons 12, 13, and 61 [2]. Patients diagnosed with mutant *KRAS* CRC have a poorer prognosis compared to those with wild-type *KRAS* CRC, particularly in the context of metastatic disease [3]. Among these mutations, the codon 12 mutation is the most prevalent, representing ~65% of *KRAS* gene alterations. Furthermore, two of the most common subtypes in CRC are the G12D (glycine at position 12 to aspartate) and G12V (glycine at position 12 to valine) mutations [4]. In addition, ~10% of CRC also contains *BRAF* V600E mutations [5]. V600E is a mutation of *BRAF*, in which valine (V) is replaced by glutamic acid (E) at position 600.

KRAS proteins activate several signaling pathways through the direct binding of effector molecules to GTP-loaded RAS. CRAF was the first effector to be discovered. Subsequent biochemical analysis revealed that CRAF performs a crucial role in signal transduction from RAS proteins to MEK and ERK kinases, together with its associated family members ARAF and BRAF [6]. Understanding the oncogenic mechanisms driven by KRAS is important, as it was considered an undruggable target. Presently, the FDA has approved two RAS inhibitors, Sotorasib and Adagrasib [7,8]. However, these inhibitors have several limitations, including their ineffectiveness in combating colorectal cancer and their lack of efficacy against other KRAS mutations [6].

There are several ongoing studies to overcome the limitations of KRAS inhibitors. Using pan-RAF inhibitors is one of the most promising methods to circumvent the limitations of KRAS inhibitors. Pan-RAF inhibitors are designed to target A-Raf, B-Raf, and C-Raf, the three members of the RAF protein family [9]. Consequently, pan-RAF targeting has become the primary treatment strategy for malignant tumors with non-G12C RAS mutations. For example, the pan-RAF inhibitor LY3009120 has demonstrated remarkable efficacy in CRC cells with different KRAS mutations, and the in vivo efficacy is also noteworthy [10]. 

Nevertheless, patients who respond to these oncogene-targeted therapies usually develop resistances to treatment and recurrences of cancer [11]. Substantial evidence indicates that resistance to RAF inhibition is caused by the formation of an immunosuppressive tumor microenvironment (TME) [12,13]. Accumulating evidence suggests that reversing the immunosuppressive TME is essential for enhancing the clinical efficacy of cancer therapy [14]. It is well-established that tumor-associated macrophages (TAMs) and other myeloid cells are associated with the immunosuppressive TME. In response to tumor-derived CSF1, immunosuppressive M2 macrophages undergo active polarization and recruitment to the TME [15,16]. CSFR1 inhibition reduces TAM infiltrators that suppress T cells [17,18]. Thus, CSF1R inhibitors have emerged as an exciting partner for T-cell-enhancing immunotherapies. In particular, the attack of tumor cells by chemotherapy-induced stimulation of tumor-derived CSF1 release, followed by an increase in TAM infiltration, provides the tumor with additional growth and survival factors. A comparable process was described for anti-angiogenic therapy, which resulted in an increase in vascular endothelial growth factor (VEGF) production by TAMs. Therefore, the combination of RAF-targeted or anti-angiogenic treatments with CSF1R inhibitors holds the potential to significantly augment antitumor efficacy [11,14]. 

We hypothesized that attaining a superior antitumor effect can be attributed to two primary factors. First, the inhibition of the MAPK pathway in RAS mutant cells, facilitated by pan-RAF inhibition, may contribute to this effect. Second, the depletion of immune suppressor cells, specifically M2 macrophages and regulatory T cells (Tregs), can be caused by the inhibition of immunokinases such as CSF1R, which may further enhance the antitumor response. Our research findings support this hypothesis, and we observed that the inhibition of pan-RAF and CSF1R using SJ-C1044, a highly potent competitive inhibitor, resulted in a significant delay in tumor growth in both cell-based and mouse models harboring mutant KRAS.

## 2. Materials and Methods

### 2.1. Molecular Modeling Study of the BRAFV600E/ SJ-C1044 Complex

The X-ray crystal structure of the V600E-BRAF (PDB ID: 5C9C) was downloaded from the Protein Data Bank in PDB format. SJ-C1044 was drawn in ChemDraw (version 12, PerkinElmer; Waltham, MA, USA). For the molecular docking analysis of SJ-C1044 with the V600E-BRAF kinase domain (PDB ID: 5C9C), we utilized Discovery Studio^®^ 2020 (BIOVIA; San Diego, CA, USA). The docking process involved a series of sequential steps, including Receptor–Ligand Interactions, Docking, Docking Optimization, and Dock Ligands. During the Dock Ligands step, the following parameter values were employed: Top Hits (10), Random Conformations (50), and Orientations to Refine (50). Subsequently, the obtained results were analyzed by comparing the CDOCKER Energy and CDOCKER Interaction Energy. The CDOCKER Energy represented the combined value of the ligand’s strain energy and the protein–ligand interaction energy. On the other hand, the CDOCKER Interaction Energy quantified the non-bonding interactions between the protein and the ligand, encompassing van der Waals energy and electrostatic interaction energy. Therefore, the CDOCKER Energy and CDOCKER Interaction Energy were assessed to evaluate the binding characteristics of the protein (V600E-BRAF)–ligand (SJ-C1044) complex.

### 2.2. Kinase Assay

Kinase profiling analysis was conducted by Eurofins (Poitiers, France). A total of 79 kinase assays were performed following standard protocols provided by Eurofins. Briefly, kinase activity was measured using the Eurofins’ KinaseProfiler radiometric assay for protein kinase assays and homogeneous time-resolved fluorescence assays for lipid and atypical kinase assays. For the radiometric assay, kinase profiling was performed by incubating a panel of kinases with the test compound SJ-C1044 at a concentration of 10 µM. The assay utilized radiolabeled phosphate as a substrate to measure kinase activity. For the homogeneous time-resolved fluorescence assay, the same panel of kinases was incubated with SJ-C1044 at the same concentration. However, in this assay, kinase activity was measured using a fluorescence-based method. For determination of the IC_50_ value, measurements were made using nine concentration points ranging from 0.001 µM to 10 µM of SJ-C1044.

### 2.3. Cell Lines and Cell Culture

HCT116, LS513, SW620, HUVECs, and HT29 cell lines were purchased from the Korean Cell Line Bank (Seoul, Korea). All cell lines were maintained in RPMI or DMEM (Gibco Inc., Billings, MT, USA) supplemented with 10% FBS (Gibco, USA) and penicillin–streptomycin in an incubator at 37 °C and 5% CO_2_. Bone marrow cells were isolated from the femur and tibia of mice using a single-pass bone marrow aspiration. Cells were cultured in RPMI medium supplemented with 10% FBS and 100 ng/mL CSF1 (Peprotech, Rocky Hill, NJ, USA) for 7 days to differentiate into bone-marrow-derived macrophages (BMDMs).

### 2.4. Cell-Based Kinase Assays

Kinase selectivity profiling in cells was carried out using the KiNativ assay developed by ActivX Biosciences (La Jolla, CA, USA). HCT 116 cells were utilized for the experiment. The cells were treated with SJ-C1044 at a concentration of 10 mmol/L for 2 h. Following the treatment, the cells were lysed, and the lysates were processed for further analysis. Probe labeling was performed using the lysates, and the labeled samples were subjected to LC/MS-MS (Liquid Chromatography/Mass Spectrometry) analysis [19].

### 2.5. Cell Proliferation Assays

In a 96-well plate, HCT 116 cells were seeded and allowed to adhere for 24 h before treatment. An SJ-C1044 dilution series was prepared and added to the wells in duplicates or triplicates. The plates were incubated for 72 h to enable cell growth and proliferation. To assess cell viability, the CellTiter-Glo Luminescent Cell Viability Assay Reagent (Promega, Fitchburg, WI, USA) was added to each well. The assay measured ATP levels as an indicator of cell viability. All data points were normalized to the control samples treated with dimethyl sulfoxide when determining IC_50_ values. Statistical calculations and data analysis, including IC_50_ determination, were performed using GraphPad Prism software.

### 2.6. Protein Immunoblots

In 6-well plates, LS513 cells were seeded and allowed to adhere and proliferate for 24 h. Subsequently, the cells were treated with SJ-C1044 for 1 h. After treatment, the cells were harvested using RIPA lysis buffer to extract cellular proteins. The cell lysates were subjected to Western blot analysis. Antibodies specific to phosphorylated epidermal growth factor receptor (p-EGFR, Cell Signaling Technology, Danvers, MA, USA, #2234S), epidermal growth factor receptor (EGFR, Cell Signaling Technology #2232S), phosphorylated vascular endothelial growth factor receptor (p-VEGFR, Cell Signaling Technology #3770S), vascular endothelial growth factor receptor (VEGFR, Cell Signaling Technology #2479S), phosphorylated Akt (p-Akt, Cell Signaling Technology #9271S), Akt (Cell Signaling Technology #9272S), phosphorylated MEK (p-MEK, Cell Signaling Technology #9121S), MEK (Cell Signaling Technology #9122), phosphorylated ERK (p-ERK, Cell Signaling Technology #9101S), and ERK (Cell Signaling Technology #9102S) were used for protein detection. The antibodies were diluted 1:1000 for the specific target proteins. In addition, a β-actin antibody (Sigma-Aldrich, Seoul, Republic of Korea, #A5441) diluted 1:5000 was used as a loading control. Bone-marrow-derived macrophages (BMDMs) were plated and allowed to adhere and proliferate for 24 h. Next, the cells were treated with SJ-C1044 for 2 h. Subsequently, the cells were stimulated with recombinant human angiopoietin-2 (R&D Systems, Minneapolis, MN, USA, #623-AN) and CSF1 (Peprotech, #300-25) for 15 min. After stimulation, protein samples were obtained from the cells. The cell lysates were analyzed by Western blot using specific antibodies, such as p-CSF1R (Cell Signaling, #14591S), CSF1R (Cell Signaling, #67455S), p-TIE2 (Cell Signaling, #4221S), and TIE2 (Santa Cruz Biotechnology, Dallas, TX, USA, #sc-293414), to detect and analyze the respective proteins.

### 2.7. Tube Formation Assays

Cell culture plates were coated with Matrigel at a concentration of 7 mg/mL and incubated at 37 °C for 45 min to allow gel formation. Human umbilical vein endothelial cells (HUVECs) were harvested using trypsin and resuspended in endothelial cell growth medium. Following a 5 h incubation period, the formation of endothelial cell tubes was assessed using an inverted photomicroscope (Nikon, Tokyo, Japan). Microphotographs were taken, and the quantification of tube formation, specifically tube length, was performed. To quantify tube formation, measurements were obtained using ImageJ. The long axis of each tube or single cell, as well as groups of adjacent cells, was measured.

### 2.8. Pharmacokinetic Studies

In the male C57BL/6 mouse model (6–8 weeks old), SJ-C1044 was administered orally (po) at a dose of 40 mg/kg and intravenously (iv) at a dose of 10 mg/kg. Blood concentrations of SJ-C1044 were measured at specific time points, including 30 min, 1 h, 2 h, 3 h, 4 h, 5 h, 6 h, and 24 h post-administration. Blood samples were collected at each time point for analysis. Plasma samples were analyzed by liquid chromatography–mass spectrometry (LC-MS/MS).

### 2.9. Animals

Six-week-old female BALB/c nude mice or C57BL/6 mice were obtained from Orient Bio and housed in individual cages at a temperature of 22 ± 3 °C and 50 ± 20% humidity. The lights were turned on and off every 12 h. The mice were provided with sterilized bedding and feed, and their water supply was first-distilled water. All experiments were conducted according to the guidance of the Institutional Animal Care and Use Committee (IACUC) at Samjin Pharmaceutical Co. (Seoul, Republic of Korea), under Project Identification Code SJ-2016-004. and the IACUC at Hanyang University (Seoul, Republic of Korea).

### 2.10. In Vivo Efficacy

Human-derived colorectal cancer cell lines, LS513, HCT-116, and HT29, were subcutaneously transplanted into 6-week-old female BALB/c nude mice to establish tumor models. Additionally, the mouse cancer cell line MC38 was subcutaneously transplanted into 6-week-old female and C57BL/6 mice to induce tumor formation. Mice with an average tumor volume of ~100 mm^3^ were selected and randomly assigned to different treatment groups, including vehicle, low (20 mg/kg), medium (40 mg/kg), and high (80 mg/kg) concentration doses of SJP1601. The treatments were administered orally once daily for 2 weeks based on the growth rate observed in the vehicle group. Tumor volume was evaluated three times a week using the formula Tumor volume (TV) = (tumor short diameter ^2^ × tumor long diameter)/2. The body weight of the mice was measured three times a week to assess potential toxicity in vivo. The tumor growth inhibition rate (% TGI) was calculated using the formula %TGI = 100 × [1 − (TVfinal treated − TVinitial treated)/(TVfinal control − TVinitial control)].

### 2.11. Immunofluorescence

Immunofluorescence staining was performed on tumor tissues. First, tumor tissue fixation was performed using 4% paraformaldehyde (Sigma-Aldrich, #HT5011). Next, heat-induced epitope retrieval was performed by incubating the tissue in 10 mM sodium citrate for 10 min. Subsequently, permeabilization was achieved by treating the tissue with 0.2% Triton X-100 (Sigma-Aldrich, #X100) for 5 min. To block nonspecific binding, the tissue was incubated in 5% skim milk for 40 min. Primary antibodies, including FOXP3 (eBioscience, San Diego, CA, USA, #11-5773-82), CD8 (eBioscience #53-0081-82), CD31 (Abcam, Cambridge, UK, #ab28364), F480 (Abcam #ab6640), CD163 (Abcam #ab182422), and CD206 (Abcam #ab64693), were used. The secondary antibodies used were FITC-conjugated AffiniPure F(ab’)2 fragment goat anti-rat IgG (H + L) (Jackson ImmunoResearch, West Grove, PA, USA, #112-096-003) and Cy3-conjugated AffiniPure F(ab’)2 fragment goat anti-rabbit IgG (H + L) (Jackson ImmunoResearch #111-166-003). The stained tissue was mounted using ProLong™ Gold Antifade Mountant with DAPI (Invitrogen, Waltham, MA, USA) #14-5773-82). Fluorescent images of the samples were captured using the Agilent BioTek Cytation 1 device. The images were analyzed using the Gen5 program (Version3, BioTek, Winooski, VT, USA) to extract relevant data and perform quantitative analysis.

### 2.12. Material

In this study, SJ-C1044 was used at a purity level exceeding 99.79%, as confirmed by high-performance liquid chromatography (HPLC). SJ-C1044 was synthesized at Samjin Pharmaceutical Co. (Seoul, Republic of Korea). For the in vivo experiments, the vehicle formulation for SJ-C1044 was prepared by combining dimethylacetamide, cremophor phosphoric acid, and 21.5% hydroxypropyl-beta-cyclodextrin in a ratio of 1:3:11, respectively. LY3009120 was purchased from Selleckchem (Houston, TX, USA)

### 2.13. Statistical Analysis

Data were analyzed using GraphPad Prism (Version8, GraphPad; San Diego, CA, USA). A two-tailed Student’s *t*-test was used to establish statistical significance.

## 3. Results

### 3.1. Discovery of Pan-RAF Inhibitor SJ-C1044

**Figure 1:**
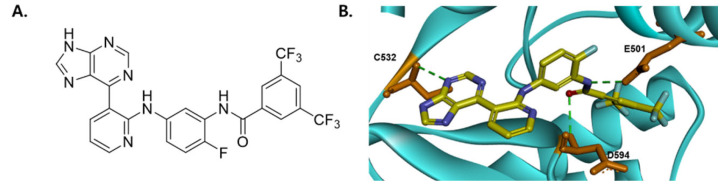
Chemical structure and binding mode of SJ-C1044 in BRAF V600E. (**A**) Chemical structure of SJ-C1044. (**B**) View of the binding mode of SJ-C1044 in BRAF V600E. SJ-C1044 and protein residues are represented as sticks with the following atom colors: carbon (SJ-C1044) yellow; carbon (BRAF V600E) brown; oxygen, red; nitrogen, blue; fluorine, cyan. The green lines with dashes indicate hydrogen bonding interactions. Molecular modeling was conducted in Discovery Studio using CHARMm.

SJ-C1044, with the chemical formula *N*-(5-(3-(9*H*-purin-6-yl)pyridin-2-ylamino)-2-fluorophenyl)-3,5-bis(trifluoromethyl)benzamide (Figure 1A), is a synthetic BRAF inhibitor designed to bind to the ATP-binding site of the BRAF(V600E) protein. Figure 1B represents the docking model for SJ-C1044 with the released X-ray crystal structure of BRAF(V600E) (PDB 5C9C). By evaluating the binding energies obtained from the docking analysis, we discerned the presence of three key residues within the BRAF protein that engaged in hydrogen bonding interactions with SJ-C1044 (Supplementary Figure S1). The purine ring of SJ-C1044 interacted with the amide backbone of CYS532 of the hinge region. Two hydrogen bonds existed between the amide oxygen and NH of SJ-C1044 and ASP594 and GLU501, respectively. These hydrogen bonding interactions supported SJ-C1044 interactions with BRAF in a DFG-out and α-C helix-in configuration [20].

### 3.2. SJ-C1044 Is a Pan-RAF Inhibitor of RAF Isoforms and Immunokinases

The biochemical activity of SJ-C1044 was characterized using recombinant proteins. In kinase assays, SJ-C1044 inhibited wild-type BRAF, wild-type CRAF, and BRAF(V600E) with IC_50_ values of 331, 257, and 187 nM, respectively (Table 1). The lack of specificity for RAF compared to other kinases was an issue that potentially restricted the clinical effectiveness of type II RAF inhibitors [10,21,22,23]. We investigated the selectivity of SJ-C1044 using two alternate screening methods. SJ-C1044 had IC_50_ values > 10 µM against 73 of 76 non-RAF kinases in biochemical assays with purified proteins. The only kinases inhibited by more than 50% were VEGFR2 (Vascular Endothelial Growth Factor Receptor 2), TIE2 (TEK Receptor Tyrosine Kinase), and CSF1R (Colony Stimulating Factor-1 Receptor) (Table 1, Supplementary Table S1). With IC_50_ values of 100, 23, and 235 nM, respectively, SJ-C1044 inhibited VEGFR2, TIE2, and CSF1R. Consistent with the results of the kinase assay, our molecular docking studies confirmed the binding of SJ-C1044 to both VEGFR2 and CSF1R (Supplementary Figures S2 and S3). Next, we analyzed SJ-C1044 using the KiNativ system, in which kinase selectivity in cells was determined by competing with an ATP-competitive covalent probe, followed by mass spectrometry analysis [24]. To evaluate the universal selectivity of SJ-C1044, we analyzed it in HCT 116 cell line lysates. LC/MS-MS analysis was employed to analyze and identify the kinase targets within the lysates, providing information on the selectivity of SJ-C1044 towards specific kinases in the cellular context. The profiling was conducted at concentrations of 10, 1, 0.1, and 0.01 µM. In HCT 116 cells incubated for 2 h with 10 µM SJ-C1044, the only kinases where probe binding was inhibited >80% were BRAF (86%) and CRAF (83%), having IC_50_ values of 1.3 and 1.4 µM, respectively (Table 2). Notably, the analysis did not include VEGFR2, TIE2, or CSF1R, as these kinases were not expressed in the HCT116 cell line.

### 3.3. SJ-C1044 Potently Inhibits KRAS-Activated MEK-ERK Phosphorylation and Cell Proliferation

**Figure 2:**
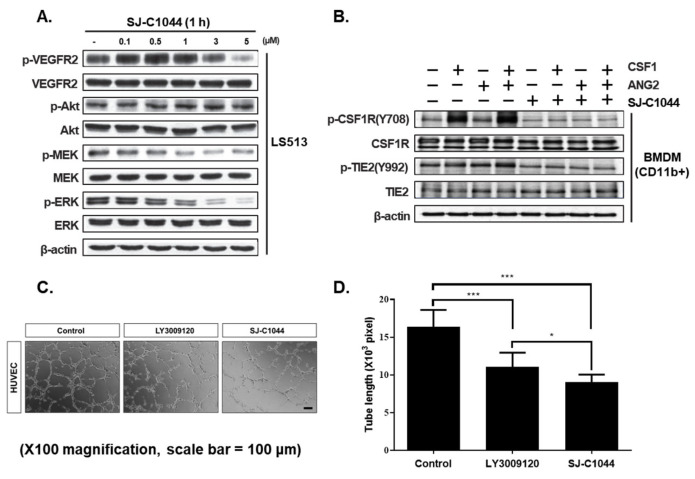
Effect of SJ-C1044 in mutant KRAS colorectal cancer cells, bone-marrow-derived macrophages (BMDMs), and human umbilical vein endothelial cells (HUVECs). (**A**) LS513 cells were treated with SJ-C1044 at the indicated concentrations. Western blot analysis was performed to evaluate the protein expression and phosphorylation of the indicated proteins. (**B**) BMDMs were treated with SJ-C1044 for 2 h, followed by stimulation with angiopoietin 2 (100 ng/mL) and CSF1 (100 ng/mL) for 15 min. Protein samples were obtained for Western blot analysis to evaluate the expression and phosphorylation status of the indicated proteins. (**C**) Tube formation induced by vascular endothelial growth factor (VEGF) in HUVECs was more effectively blocked by SJ-C1044 compared to LY3009120. (**D**) HUVECs were incubated with a concentration of 10 μM of LY3009120 or SJ-C1044, along with 10 nmol/L VEGF. Data are expressed as mean tube length ± SEM (*n* = 5). * *p* < 0.05; *** *p* < 0.001 (Student’s *t*-test, two-tailed).

The effect of SJ-C1044 on oncogenic KRAS-mediated MEK and ERK phosphorylation was investigated in LS513, a KRAS(G12D)-mutant colorectal cancer cell line (Figure 2A). SJ-C1044 inhibited MEK and ERK phosphorylation potently, which was consistent with the biochemical characterization data. There was no detectable difference in AKT phosphorylation. Interestingly, VEGFR2 was also expressed in LS513, and SJ-C1044 inhibited VEGFR2 activity at the cellular level. We found that SJ-C1044 effectively inhibited the receptor kinase activity of VEGFR2 in colorectal cancer cell lines. These findings highlighted the ability of SJ-C1044 to modulate VEGFR2 activity in the context of colorectal cancer. To test its potency against KRAS and BRAF mutant colorectal cancer cells, we treated three KRAS-mutant cancer cell lines (HCT116, LS513, and SW620) or a BRAF-mutant cancer cell line (HT29) with SJ-C1044 in a cell growth assay. Our results showed that SJ-C1044 impaired the growth of all KRAS and BRAF mutant cancer cells, with IC_50_ values ranging from 160 to 628 nM (Table 3). These results confirmed that SJ-C1044 induced cytotoxicity in KRAS-mutated cancer cells in vitro.

### 3.4. Potent Inhibition of TAM Signaling and Angiogenesis by SJ-C1044

SJ-C1044, a potent inhibitor of CSF1R and TIE2, displayed significant inhibitory effects on the ligand-dependent phosphorylation of CSF1R and TIE2 in BMDMs (Figure 2B). Furthermore, SJ-C1044 demonstrated strong activity against VEGF-stimulated endothelial tube formation, indicative of its potent anti-angiogenic properties through inhibition of VEGFR2 (Figure 2C,D). SJ-C1044 inhibited the formation of the tube to a greater degree compared to LY3009120. These findings highlight the potential of SJ-C1044 as a multi-targeting therapeutic agent with the ability to inhibit angiogenesis in endothelial cells and disrupt crucial cellular signaling pathways in TAMs.

### 3.5. Pharmacokinetic Studies of SJ-C1044

**Figure 3:**
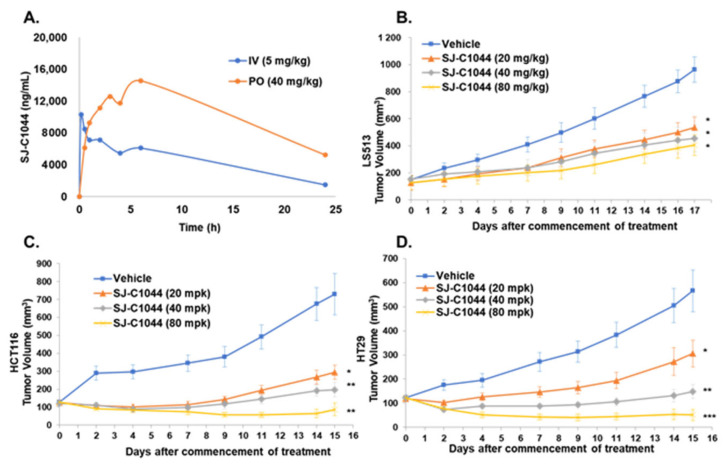
Antitumor activity of SJ-C1044 in xenograft models of mutant KRAS or mutant BRAF. (**A**) Plasma concentration-time profile of SJ-C1044 in mice. SJ-C1044 was administered orally (po) at a dose of 40 mg/kg and intravenously (iv) at a dose of 5 mg/kg to male C57BL/6 mice. The blood concentrations of SJ-C1044 were measured at 30 min, 1 h, 2 h, 3 h, 4 h, 5 h, 6 h, and 24 h post-administration. (**B**–**D**) Anti-tumor growth activities of SJ-C1044 in LS513 (**B**), HCT116 (**C**), and HT29 (**D**) mouse xenograft models. SJ-C1044 was orally administered once daily (QD) at doses of 20, 40, or 80 mg/kg. Vehicle, blue line; SJ-C1044 20 mg/kg QD, red line; SJ-C1044 40 mg/kg QD, gray line; SJ-C1044 80 mg/kg QD, orange line. Error bars represent ± SEM (*n* = 10). *** *p* < 0.001; ** *p* < 0.01; * *p* < 0.05 (Student’s *t*-test, two-tailed).

The pharmacokinetics of SJ-C1044 were evaluated in C57BL/6 mice. The study involved oral (po) and intravenous (iv) administration of SJ-C1044 at doses of 40 mg/kg and 5 mg/kg, respectively. Blood samples were collected at various time intervals up to 24 h post-administration to measure the concentrations of SJ-C1044. Following po administration, the mean peak plasma concentration (*C_max_*) reached 14,885 ng/mL, with a mean time to *C_max_* (*T_max_*) of 4.3 h. In the case of iv administration, the corresponding values were 11,408 ng/mL for *C_max_* and 1.6 h for *T_max_* (Table 4). Plasma concentration/time curves obtained from an acute dose pharmacokinetic study in mice revealed that SJ-C1044 exhibited sustained plasma concentrations at or above 8000 ng/mL (14 μM) for 16 h per day when administered at a dose of 40 mg/kg once daily. The oral bioavailability of SJ-C1044 was determined to be 23%. These findings suggested that SJ-C1044 achieved and maintained therapeutically relevant plasma concentrations, supporting its potential efficacy as a treatment option (Figure 3A).

### 3.6. SJ-C1044 Demonstrates Antitumor Activity in KRAS or BRAF Mutant Xenograft Models of Colorectal Cancer

In the KRAS G12D LS513 colorectal cancer model, SJ-C1044 was administered orally at 20, 40, or 80 mg/kg, once a day (QD), and significant tumor growth suppression was observed in a dose-dependent manner (Figure 3B). The tumor growth inhibition (TGI) at the end of the 17th day was 50%, 63%, and 66% (*p* < 0.05) for the 20, 40, or 80 mg/kg dose group administered SJ-C1044, respectively (Table 5). Similarly, in the KRAS G13D HCT116 colorectal tumor model, SJ-C1044 administered at 20, 40, or 80 mg/kg QD showed dose-dependent tumor growth inhibition. In the HCT116 colorectal tumor model, treatment with SJ-C1044 for 16 days produced a significant growth inhibitory effect ranging from 72% to 107% (Table 5). The in vivo efficacy of SJ-C1044 was examined in xenograft models derived from HT29 colorectal cancer cell lines carrying the BRAFV600E mutation (Figure 3C). A clear dose response was observed in this model. Treatment of HT29 tumor xenografts with SJ-C1044 resulted in TGI with a range of 58% to 117% (Table 5). The results of these in vivo efficacy analyses suggested that SJ-C1044 inhibited the growth of tumors with KRAS or BRAF mutations. 

### 3.7. SJ-C1044 Increased T-Cell Infiltration While Reducing the Proportion of TAMs

**Figure 4:**
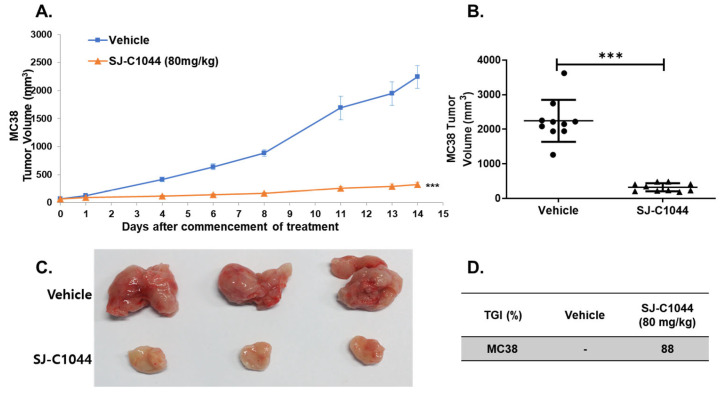
Impact of SJ-C1044 in syngeneic mouse models. (**A**) Anti-tumor growth activities of SJ-C1044 in MC38 syngeneic mouse models. SJ-C1044 was orally administered once daily (QD) at doses of 80 mg/kg. Vehicle, blue line; SJ-C1044 80 mg/kg QD, red line. Error bars represent ± SEM (*n* = 10). *** *p* < 0.001 (Student’s *t*-test, two-tailed). (**B**) Each dot represents the mean ± SEM of the tumor volume of the two groups. (**C**) Representative images showing the tumor burden acquired from each group. (**D**) TGI after 14 days of treatment with 80 mg/kg SJ-C1044.

**Figure 5:**
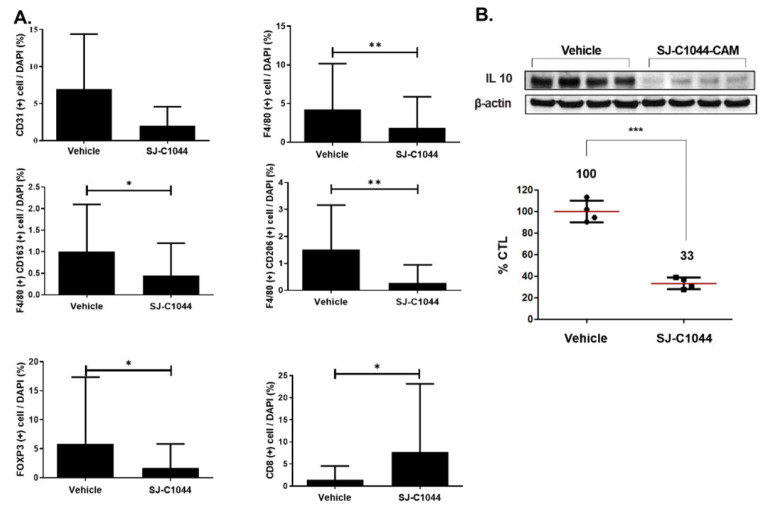
Effects of SJ-C1044 on angiogenesis, macrophages, and immune cell infiltration. (**A**) SJ-C1044 treatment reduced CD31 staining of tumor tissue, indicating an inhibitory effect on angiogenesis. SJ-C1044 decreased F4/80+ staining of tumor tissue, suggesting suppressed macrophage infiltration or activation (upper panel). SJ-C1044 administration caused a decrease in CD163+ or CD206+ staining, which are markers for M2 macrophages (middle panel). SJ-C1044 increased the infiltration of CD8+ T cells (bottom panel) and a simultaneous reduction in FOXP3+ cell population, including immunosuppressive regulatory T (Treg) cells, within the tumors. All staining intensities were normalized relative to the DAPI signals of the corresponding samples. Error bars represent ± SEM (*n* = 5). ** *p* < 0.01; * *p* < 0.05 (Student’s *t*-test, two-tailed). (**B**) SJ-C1044 treatment notably decreased IL-10 levels. Western blot analysis was performed to evaluate the protein expression of IL-10. Four independent Western blots were quantified using ImageJ. The protein expression of IL-10 was normalized relative to the β-actin signals of the corresponding samples. Mean ± SEM, *** *p* < 0.001 (Student’s *t*-test, two-tailed).

To investigate the immunomodulatory properties of SJ-C1044, the MC38 syngeneic mouse model was used. Due to their intact immune systems, syngeneic mice are particularly useful for immunotherapeutic analyses. Compared to the control, SJ-C1044 completely inhibited MC38 tumor growth in vivo (Figure 4). In the immunofluorescence analysis of the tumor tissue, we observed that CD31 staining, an endothelial cell (EC) marker to detect angiogenesis intensity, was reduced in mice treated with SJ-C1044, indicating an inhibitory effect on angiogenesis (Figure 5A, upper panel). Additionally, F4/80+ immunostaining, which served as a pan macrophage marker, exhibited high intensity within the lesions of the control group. However, in mice treated with SJ-C1044, the F4/80+ staining intensity was notably reduced, suggesting the suppression of macrophage infiltration or activation (Figure 5A, upper panel). Furthermore, the mice treated with SJ-C1044 showed a significant decrease in CD163+ or CD206+ staining, which are markers for M2 macrophages (Figure 5A, middle panel). These results provided evidence of the effective depletion of M2 macrophages by SJ-C1044 treatment. Furthermore, treatment with SJ-C1044 caused an increase in the infiltration of CD8+ T cells (Figure 5A, bottom panel) while simultaneously reducing the population of FOXP3+ cells, which included immunosuppressive cells such as Tregs within the tumors. Interestingly, treatment with SJ-C1044 resulted in a notable decrease in IL-10 levels, which is an immunosuppressive cytokine released by TAMs (Figure 5B). These findings collectively demonstrated the potential of SJ-C1044 to modulate immune cell activity in vivo. The tolerability of SJ-C1044 was thoroughly assessed in all preclinical pharmacology studies. A single oral dose toxicity study in Sprague Dawley rats at 500 mg/kg showed no respiratory effects or signs of toxicity. Similarly, a 2-week repeated oral DRF (Dose Range Finding) study in Sprague Dawley rats at 500 mg/kg revealed no indications of toxicity. In ICR mice, a single oral dose of SJ-C1044 at 500 mg/kg did not result in any abnormal changes in the central nervous system. In Beagle Dogs, a 2-week repeated oral DRF toxicity study demonstrated no toxicity at 250 mg/kg, although decreased body weight was observed at 500 mg/kg. Based on these findings, further 4-week repeated oral toxicity studies will be conducted at various dose levels to assess safety. Overall, these preclinical studies indicated a favorable tolerability profile of SJ-C1044 within the tested dose ranges in the respective animal models. In the mouse xenograft experiments described in this paper, no signs of toxicity or significant weight loss were observed when SJ-C1044 was administered.

## 4. Discussion

The results presented in this study demonstrate the potential of SJ-C1044 as a novel pan-RAF inhibitor with promising therapeutic implications for cancer treatment. The discovery of SJ-C1044, its characterization, and the evaluation of its pharmacological properties provide valuable insights into its mechanism of action and potential clinical applications.

One of the key findings of this study is the potent inhibitory activity of SJ-C1044 against BRAF (V600E) as well as wild-type BRAF and CRAF. This broad-spectrum inhibitory activity is crucial considering the involvement of these RAF isoforms in tumors containing a RAS mutation. RAF inhibitors can be classified based on the conformation they stabilize in their target kinase. “αC-IN” inhibitors stabilize the αC-helix in the IN position (either αC-helix-IN/DFG-IN for type I or αC-helix-IN/DFG-OUT for type IIa), whereas “αC-OUT” inhibitors commonly stabilize the αC-helix in the OUT position, often occurring as αC-helix-OUT/DFG-IN for type IIb, which includes clinical RAF inhibitors such as vemurafenib and dabrafenib [20]. Through molecular modeling, it was determined that SJ-C1044 establishes hydrogen bonds with GLU501, CYS532, and ASP594 of BRAF. This observation classifies SJ-C1044 as an αC-helix-IN/DFG-OUT (type IIa) BRAF inhibitor [25]. Conversely, Vemurafenib exclusively forms a hydrogen bond with ASP594 of BRAF [26]. Therefore, SJ-C1044 shares the common feature of hydrogen bonding with ASP594 of BRAF while additionally forming hydrogen bonds with GLU501 and CYS532 of BRAF, thus qualifying it as a type IIa BRAF inhibitor. The ability of SJ-C1044 to interact with BRAF (V600E) in a DFG-out and α-C helix-in configuration further highlights its specificity and potential to disrupt the aberrant MAPK signaling pathway as a type IIa RAF inhibitor. Recently, it was established that the mode of inhibitor binding plays an essential role in determining the occurrence of paradoxical activation [27]. It is believed that Type IIb inhibitors, such as vemurafenib and dabrafenib, primarily bind to BRAF monomers and effectively inhibit BRAF V600E, which can activate signaling pathways in its monomeric state [28]. However, the administration of type IIb inhibitors to KRAS mutant cancers results in the activation of the MAPK pathway within these tumors, eventually leading to the occurrence of paradoxical activation [29]. Conversely, type IIa RAF inhibitors, which bind to a DFG-out and α-C-helix-in conformation, lack the propensity for inducing paradoxical activation in KRAS mutant cells [30]. Indeed, treatment of KRAS-mutated colorectal cancer cells with SJ-C1044, a type IIa RAF inhibitor, resulted in the inhibition of MEK and ERK activity in a concentration-dependent manner, without any evidence of paradoxical activation (Figure 2A). Moreover, in vivo animal efficacy studies conducted in xenograft models of mutant KRAS demonstrated that SJ-C1044 treatment led to a dose-dependent reduction in cancer tissue growth, without the development of drug resistance (Figure 3). These findings highlight the complete inhibition of the MAPK pathway and the subsequent suppression of cancer cell growth and proliferation by SJ-C1044 in colorectal cancer cells harboring mutant KRAS, distinguishing it from Type IIb inhibitors that may exhibit reduced efficacy due to paradoxical activation.

From a therapeutic standpoint, the absence of effectiveness against ARAF may be either an advantage or a disadvantage. ARAF is highly expressed in the majority of human tissues, frequently associated with other RAF family members [21]. A complete blockade of signaling through the RAS/RAF/MEK kinase cascade is associated with poor tolerance. Therefore, the absence of ARAF activity upon SJ-C1044 administration may present a more favorable therapeutic opportunity in comparison to other pan-RAF inhibitors. As a result, the expansion of this therapeutic window holds significant potential for improved treatment outcomes [22]. Importantly, the selectivity profile of SJ-C1044 was thoroughly assessed using biochemical assays and the KiNativ system, which demonstrated minimal inhibition of ARAF and non-RAF kinases. This selectivity is crucial for minimizing off-target effects and potential toxicity. 

Our results also highlight the potent inhibitory effects of SJ-C1044 on KRAS-activated MEK-ERK phosphorylation and cell proliferation. The selective cytotoxicity observed in KRAS-mutant and BRAF-mutant cancer cells further supports the potential therapeutic utility of SJ-C1044 in targeting specific oncogenic mutations. These findings provide a strong rationale for further exploration of SJ-C1044 as a targeted therapy for colorectal cancer and other malignancies harboring KRAS or BRAF mutations.

Notably, the inhibition of VEGFR2, TIE2, and CSF1R by SJ-C1044 suggests its potential as both an angiogenesis inhibitor and a modulator of macrophage function, which can have significant implications in the TME. The observed immunomodulatory properties of SJ-C1044 in the MC38 syngeneic mouse model provide valuable insights into its ability to modulate the tumor immune microenvironment. Additionally, the results of the immunofluorescence analysis revealed notable effects of SJ-C1044 on TAMs, further supporting its impact on the immune landscape within the tumor. The reduced intensity of F4/80+ immunostaining, a pan macrophage marker, in SJ-C1044-treated mice indicated the suppression of macrophage infiltration or activation within the tumor lesions. This effect was further supported by the decreased staining intensity of CD163 and CD206, markers associated with M2 macrophages known for their immunosuppressive functions. The depletion of M2 macrophages by SJ-C1044 treatment suggests a shift towards an anti-tumor immune response within the tumor microenvironment. In addition, SJ-C1044 treatment increased CD8+ T cell infiltration, indicating an enhanced anti-tumor immune response. Simultaneously, there is a reduction in the population of FOXP3+ cells, including Tregs responsible for immunosuppression. These findings suggest that SJ-C1044 promotes an immune cell composition within the tumor that favors an anti-tumor response and attenuates immunosuppressive mechanisms. The observed decrease in IL-10 levels, an immunosuppressive cytokine released by TAMs, further supports the immunomodulatory effects of SJ-C1044. By reducing IL-10 levels, SJ-C1044 counteracts the immunosuppressive tumor microenvironment, facilitating an immune response against the tumor. Manipulating intratumoral immunity holds significant promise as a strategic approach to overcome resistance and enhance the effectiveness of RAF inhibitors in the treatment of cancer patients [11]. The findings from our study not only offer novel opportunities for concurrent inhibition but also emphasize the potential of synergistic targeting that encompasses both tumor cells and the immune system.

The favorable tolerability profile observed in the preclinical pharmacology studies, with no signs of toxicity or significant weight loss, is promising for the clinical development of SJ-C1044. However, further investigations are warranted to fully understand the safety profile and potential adverse effects of SJ-C1044 in clinical settings.

Collectively, the results of this study provide strong evidence for the therapeutic potential of SJ-C1044 in RAS or BRAF mutant colorectal cancers. By targeting multiple kinases, SJ-C1044 demonstrates its ability to reshape the tumor microenvironment and promote anti-tumor immune responses. These findings contribute to our understanding of the mechanisms underlying the anti-tumor activity of SJ-C1044 and provide a basis for further investigations and clinical developments of this multi-kinase inhibitor as an effective treatment option for RAS or BRAF mutant cancers.

## Figures and Tables

**Table 1:** Biochemical IC50 determinations for SJ-C1044 compared to a panel of kinases.

Kinase (% Inhibition)	SJ-C1044 (10 μM)	Kinase (IC_50_, nM)	SJ-C1044
ARAF	44	ARAF	>10,000
BRAF	68	BRAF	331
BRAF (V600E)	80	BRAF (V600E)	187
CRAF	86	CRAF	257
VEGFR2	91	VEGFR2	100
TIE2	97	TIE2	23
CSF1R	70	CSF1R	235

**Table 2:** Binding affinities of SJ-C1044 to ARAF, BRAF, and CRAF full-length proteins in HCT116.

Binding Affinities in HCT116 (EC_50_, μM)	SJ-C1044
ARAF	>10
BRAF	1.3
CRAF	1.4

**Table 3:** Cellular mean GI50 determinations for SJ-C1044 compared to a panel of cell lines.

Cell Line	HCT116 (KRAS^G13D^)	LS513 (KRAS^G12D^)	SW620 (KRAS^G12V^)	HT29 (BRAF^V600E^)
SJ-C1044 (GI_50_, nM)	160	290	628	247

**Table 4:** Pharmacokinetic Parameters for SJ-C1044.

	po (40 mg/kg)	iv (5 mg/kg)
AUC_last_ (ng·h/mL)	228,373	97,751
C_max_(ng/mL)	14,885	11,408
T_max_ (h)	4.3	1.6
Bioavailability (%)	23	-

**Table 5:** TGI for SJ-C1044.

TGI (%)	SJ-C1044 (20 mg/kg)	SJ-C1044 (40 mg/kg)	SJ-C1044 (80 mg/kg)
LS513 (KRAS^G12D^)	50	63	66
HCT116 (KRAS^G13D^)	72	87	107
HT29 (BRAF^V600E^)	58	94	117

## Data Availability

The data that support the findings of this study are available on request from the corresponding author Soon Kil Ahn.

## Supplementary Materials

The following supporting information can be downloaded at: https://www.mdpi.com/article/10.3390/cimb45070371/s1, Figure S1: Binding Modes of SJ-C1044 in BRAF V600E (PDB Code: 5C9C), Figure S2: Binding Modes of SJ-C1044 in VEGFR2 (PDB Code: 3CP9), Figure S3: Binding Modes of SJ-C1044 in CSF1R (PDB Code: 3KRL), and Table S1: Kinase assays of SJ-C1044.
