# Supplementary material for: Targeting RAF Isoforms and Tumor Microenvironments in RAS or BRAF Mutant Colorectal Cancers with SJ-C1044 for Anti-Tumor Activity
Source: Curr Issues Mol Biol. 2023 Jul 13;45(7):5865–78. doi: 10.3390/cimb45070371 (PMC10378394; doi:10.3390/cimb45070371)
Supplement: Supplementary file 1 [file cimb-45-00371-s001.zip › cimb-2460475-supplementary.pdf]

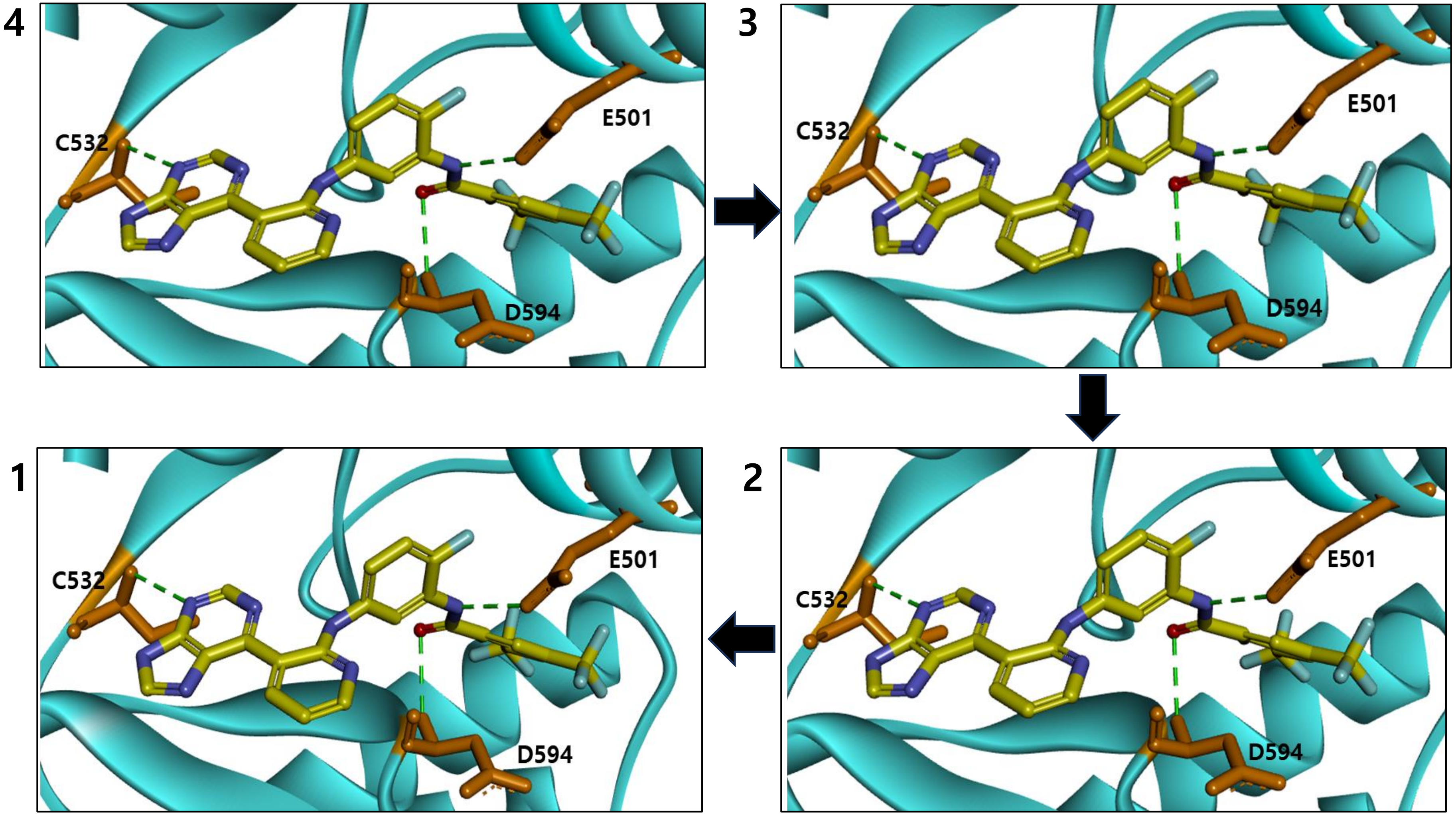

Binding Mode in BRAFV600E	CDOCKER Energy	CDOCKER Interaction Energy
**4**	-28.1283	-63.1195
**3**	-28.1671	-62.589
**2**	-28.5095	-63.1158
**1**	**-28.7647**	**-63.2025**

Supplementary Figure S1. Binding Modes of SJ-C1044 in BRAF V600E (PDB Code: 5C9C).

The binding modes of SJ-C1044 and BRAF V600E were determined using Discovery Studio software. The binding energy was subsequently calculated and optimized. Among the identified binding modes, the predicted binding mode is designated as mode 1 due to its lowest CDOCKER Energy value.

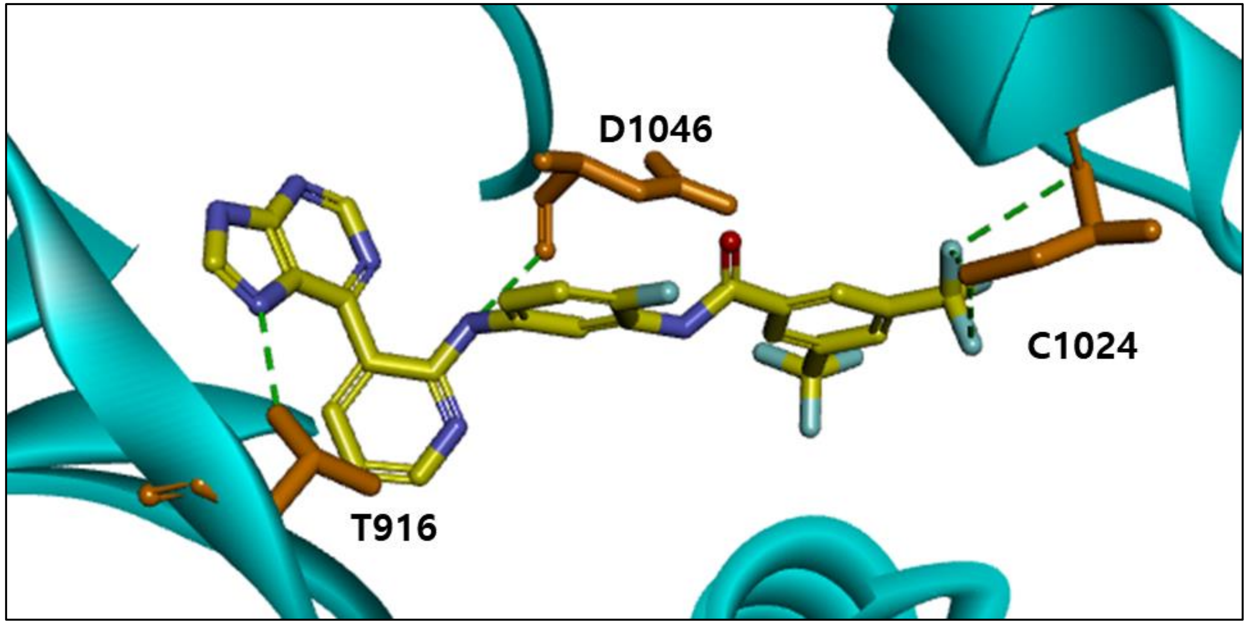

Binding Mode in VEGFR2	CDOCKER Energy	CDOCKER Interaction Energy
	-18.9631	-54.6837

Supplementary Figure S2. Binding Modes of SJ-C1044 in VEGFR2 (PDB Code: 3CP9) The binding modes of SJ-C1044 and VEGFR2 was determined using Discovery Studio software. The binding energy was subsequently calculated and optimized

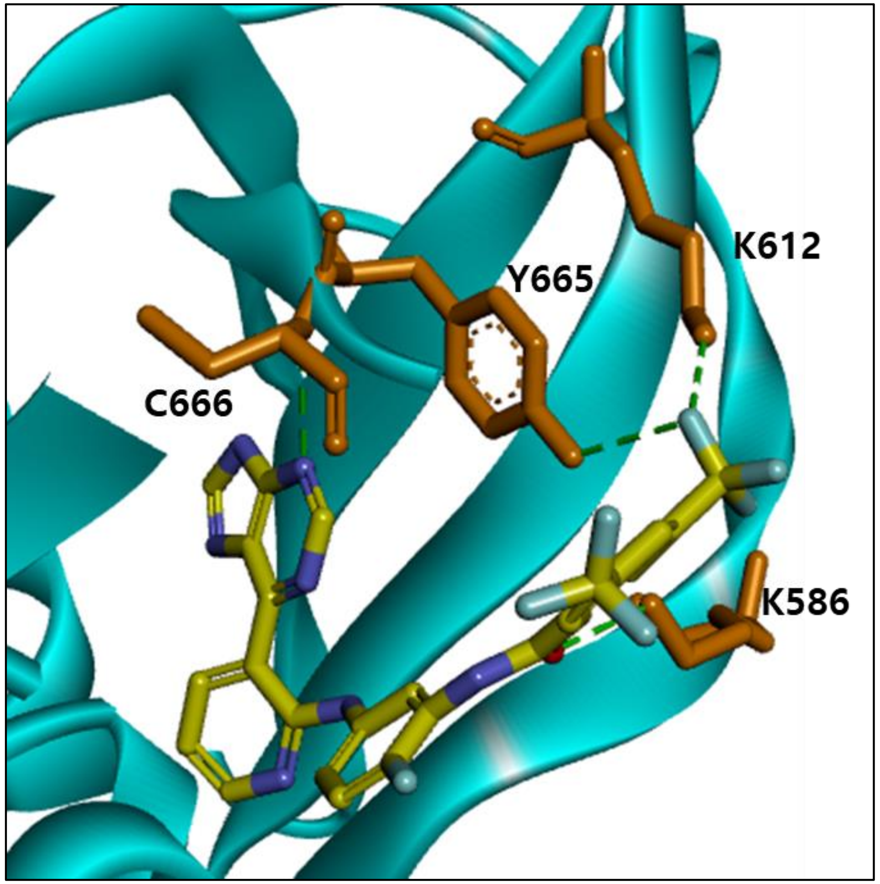

Binding Mode in CSF1R	CDOCKER Energy	CDOCKER Interaction Energy
	-18.426	-51.4336

Supplementary Figure S3. Binding Modes of SJ-C1044 in CSF1R (PDB Code: 3KRL) The binding modes of SJ-C1044 and CSF1R was determined using Discovery Studio software. The binding energy was subsequently calculated and optimized

Supplementary Table S1

	SJ-C1044 @ 10 μM	Kinase (% inhibition)
1	TIE2	97
2	VEGFR2	91
3	c-RAF(h)	86
4	B-Raf(V600E)(h)	80
5	CSF1R(h)	70
6	B-Raf(h)	68
7	FGFR3(h)	48
8	A-Raf(h)	44
9	cSRC(h)	38
10	PI3Kinase(p110a(H10	37
11	Fyn(h)	27
12	PI3Kinase(p110a(E54	24
13	PI3Kinase(p110a/p85	23
14	PI3Kinase(p110a(E54	20
15	BTK(h)	18
16	Yes(h)	17
17	PKBα(h)	15
19	Met(h)	13
20	ATM(h)	12
21	PDGFRβ(h)	11
22	PKCι(h)	11
23	CDK7/cyclinH/MAT1(	10
24	PKCβII(h)	10
25	MST2(h)	9
26	p70S6K(h)	8
27	ATR/ATRIP(h)	8
28	PKCθ(h)	5
29	CK1(y)	4
30	Rsk3(h)	4
31	Aurora-A(h)	3
32	CSK(h)	3
33	Flt3(h)	3
34	PAR-1Bα(h)	3
35	PKCη(h)	3
36	PKD2(h)	3
37	CaMKIV(h)	2
38	CDK2/cyclinE(h)	2
39	PKBγ(h)	2
40	MEK1(h)	1
41	PKBβ(h)	1
42	PKCγ(h)	1
43	PKCζ(h)	1
